# Interplay between Endoplasmic Reticular Stress and Survivin in Colonic Epithelial Cells

**DOI:** 10.3390/cells7100171

**Published:** 2018-10-15

**Authors:** Rohit Gundamaraju, Ravichandra Vemuri, Wai Chin Chong, Stephen Myers, Shaghayegh Norouzi, Madhur D. Shastri, Rajaraman Eri

**Affiliations:** School of Health Sciences, University of Tasmania, Launceston, TAS 7248, Australia; ravichandra.vemuri@utas.edu.au (R.V.); chongwc1993@gmail.com (W.C.C.); Stephen.myers@utas.edu.au (S.M.); shaghayeg.norouzi@utas.edu.au (S.N.); madhur.shastri@utas.edu.au (M.D.S.)

**Keywords:** endoplasmic reticular stress, apoptosis, survivin, unfolded protein response, inhibition of apoptosis, proliferation, Winnie, LS174T, colon, colon cancer, 4PBA, tunicmycin

## Abstract

Sustained endoplasmic reticular stress (ERS) is implicated in aggressive metastasis of cancer cells and increased tumor cell proliferation. Cancer cells activate the unfolded protein response (UPR), which aids in cellular survival and adaptation to harsh conditions. Inhibition of apoptosis, in contrast, is a mechanism adopted by cancer cells with the help of the inhibitor of an apoptosis (IAP) class of proteins such as Survivin to evade cell death and gain a proliferative advantage. In this study, we aimed to reveal the interrelation between ERS and Survivin. We initially verified the expression of Survivin in Winnie (a mouse model of chronic ERS) colon tissues by using immunohistochemistry (IHC) and immunofluorescence (IF) in comparison with wild type Blk6 mice. Additionally, we isolated the goblet cells and determined the expression of Survivin by IF and protein validation. Tunicamycin was utilized at a concentration of 10 µg/mL to induce ERS in the LS174T cell line and the gene expression of the ERS markers was measured. This was followed by determination of inflammatory cytokines. Inhibition of ERS was carried out by 4Phenyl Butyric acid (4PBA) at a concentration of 10 mM to assess whether there was a reciprocation effect. The downstream cell death assays including caspase 3/7, Annexin V, and poly(ADP-ribose) polymerase (PARP) cleavage were evaluated in the presence of ERS and absence of ERS, which was followed by a proliferative assay (EdU click) with and without ERS. Correspondingly, we inhibited Survivin by YM155 at a concentration of 100 nM and observed the succeeding ERS markers and inflammatory markers. We also verified the caspase 3/7 assay. Our results demonstrate that ERS inhibition not only significantly reduced the UPR genes (*Grp78*, *ATF6*, *PERK* and *XBP1*) along with Survivin but also downregulated the inflammatory markers such as IL8, IL4, and IL6, which suggests a positive correlation between ERS and the inhibition of apoptosis. Furthermore, we provided evidence that ERS inhibition promoted apoptosis in LS174T cells and shortened the proliferation rate. Moreover, Survivin inhibition by YM155 led to a comparable effect as that of ERS inhibition, which includes attenuation of ERS genes and inflammatory markers as well as the promotion of programmed cell death via the caspase 3/7 pathway. Together, our results propose the interrelation between ERS and inhibition of apoptosis assigning a molecular and therapeutic target for cancer treatment.

## 1. Introduction

The disruption of endoplasmic reticular homeostasis by various physiological stimuli leads to the accumulation of misfolded proteins. This phenomenon is called endoplasmic reticular stress (ERS), which, in turn, induces a coordinated program called an unfolded protein response [1]. The unfolded protein response (UPR) triggered by ERS serves as an adaptive mechanism to protect the cell from stress and restore endoplasmic reticulum (ER) homeostasis. An increase in ERS initiates the UPR, which results in programmed cell death or apoptosis. In conditions such as cancer, ERS initiates an unfavorable micro-environment that may include low pH and hypoxia, which leads to the accumulation of misfolded proteins. Under normal cellular conditions, the above factors collectively lead to apoptosis to ameliorate ER stress and reinstate homeostasis. In cancer, numerous mechanisms have evolved to circumvent environmental stress and evade cell death [2,3].

ER chaperones are the group of proteins typically involved in shuttling proteins into ER and modifying with *N*-linked glycans, which was folded into the appropriate secondary and tertiary structures. These are stabilized by disulfide bonds and, in many cases, are assembled into multimeric complexes [4]. Inhibition of ER chaperone proteins that aid in correct protein folding has been suggested as a therapeutic approach in cancer treatment, which targets pro-survival pathway inhibition and subsequent initiation of cancer cell death [5]. Apoptosis is dependent on the triggering of caspases and a special class of proteins known as an inhibitor of apoptosis proteins (IAPs), which negatively regulates caspases and apoptosis [6]. Numerous cellular processes are controlled by IAPs. In cancers, IAPs either directly or indirectly contribute to tumorigenesis through disease initiation, progression, cellular proliferation, and tumor preservation. Thus, IAPs are gaining clinical interest for their role in therapeutics as tumor markers.

Survivin is one of the IAP that protects cells from apoptosis. Survivin was reported to act as a ‘gatekeeper’ at the G2-M boundary of the cell cycle and resides between the nucleus and cytoplasm, which binds to caspase activation involved in apoptosis [7]. Survivin is highly organ-specific and tissue-specific and is linked to several processes such as angiogenesis. The stress response and p53 linkage as Survivin was said to regulate p53 [8]. Survivin is responsible for aggressiveness cellular proliferation in colorectal cancers (CRC) and its inhibition via PARP6 resulted in the reduction of tumors [9]. This highlights an essential role for survivin as a critical regulator of tumorigenesis and is, therefore, amendable to therapeutic intervention in the treatment of cancer progression.

ER stress in situ often correlates with advanced-stage disease and chemoresistance. The ability to tolerate persistent ER stress enhances cancer cell survival, angiogenesis, metastatic capacity, drug resistance, and immune-suppression [10]. ERS inhibition has gained attention in establishing a therapeutic approach to treat various disease states. ERS inhibitors including benzodiazepines, baicalein, 1-deoxymannojirimycin hydrochloride, and others targeting ERS genes such as *CHOP* and ER alpha-mannosidase were employed in various cell lines including stem cells and progenitor cells to bring down the levels of reactive oxygen species (ROS) and chaperones in order to establish a therapeutic role [11]. ER misfolding defects are associated with aggressive tumor growth and, therefore, it is critical to understand the molecular mechanisms and regulation of the UPR. Cancer survival in one way depends on the UPR signaling pathways that orchestrate cellular processes such as apoptosis and autophagy. Pharmacological induction of ERS leads to escalation of UPR markers and pro-inflammatory cytokines [12]. In addition, ERS inducers such as thapsigargin in tumor mice aggravated the tumors, which form a link between ERS and cancer progression. However, there was no clear link between ERS and IAP proteins such as Survivin and, hence, we decided to investigate the relationship between these two mechanisms and subsequent downstream effects like inflammation, apoptosis, and proliferation.

In the present study, we determined the expression of Survivin in Winnie, which is a chronic ERS mouse model displaying severe colitis due to missense *Muc2* mutations [13]. We have also correlated *Muc2* expression with proliferation in LS174T cells since the *Muc2* role was perplexing in the gut due to a number of studies correlating *Muc2* expression with severe ERS [14] and *Muc2* positive expression, which was relatable with increased proliferation [15].

Importantly, Survivin expression is a well-established event in the development of colonic adenocarcinoma [16]. Studies have documented Survivin translocation between the nucleus and cytoplasm. Its potential role as an inhibitor of apoptosis is conducted by binding to the mitochondrial activator of caspase and portraying it as a bridge between apoptosis and mitosis [17]. Apoptosis and ERS, in contrast, are responsible for the development of various diseases. The molecular link between ERS and apoptosis has not yet been established based on a plethora of complex events including the accumulation of folded proteins and hypoxia as part of the pro-survival mechanism [18]. Hence, it was vital to understand the link between the inhibition of apoptosis, ERS, pro-survival, proliferation, and cancer.

We have shown for the first time a reciprocal relationship between ERS and Survivin through chemical inducers and inhibitors of ERS and Survivin activity. This relationship was also concomitant with cell death and the rate of cellular proliferation in the human colon cancer cell line LS174T.

## 2. Materials and Methods

### 2.1. Cell Culture

The human colon cancer cell line (LS174T-ATCC^®^ CL-188™) was cultured in Roswell Park Memorial Institute medium RPMI media with added l-glutamine (Life Technologies, Victoria, Australia) supplemented with 10% fetal bovine serum, penicillin (1000 UG/mL), and streptomycin (1000 U/mL) (Gibco BRL, Victoria, Australia). Cells were incubated under 37 °C and 5% CO_2_. After reaching confluency, the cells were harvested using 0.25% TrypLe express (Life Technologies, AUS). The detached cells were determined for the cell number and viability after washing by using the Countess^®^ cell counter (Life Technologies, AUS) as per the instructions.

### 2.2. Animals

All animal experiments were approved by the Animal Ethics Committee of the University of Tasmania (Ethics approval number: A14095, 2017) and conducted in accordance with the Australian Code of Practice for Care and Use of Animals for Scientific Purposes (8th Edition 2013). All the animals were bought from the Cambridge farm facility (Hobart, Australia). Animal housing was under a 21 °C environment with a 12-h day/night cycle. All mice had continuous access to radiation-sterilized rodent feed (Barastoc Rat and Mouse, Ridley AgProducts, Hobart, Australia) and autoclaved tap water ad libitum.

### 2.3. Isolation of Goblet Cells

The animals were sacrificed via CO_2_ asphyxiation in the morning before the abdomen was dissected and the colon removed. Colons were then harvested from 16 weeks, 10 Winnie (homozygous *Muc2* mutant), and 10 wild type mice (C57BL6/J background) of both sexes, sliced lengthwise, and washed with ice cold phosphate buffer saline (PBS). The colons were stored on ice in Dulbeccos’ Modified Eagle Medium (DMEM) l-glutamine (Life Technologies) and supplemented with 10% fetal bovine serum (Gibco, VIC, AUS), penicillin (1000 U/mL), and streptomycin (1000 ug/L) (Gibco BRL, AUS) until all colons were collected. The colons were washed twice with PBS and placed in pre-digestion solution (liberase 0.35 mL; Hanks’ balanced salt solution HBSS). The liberase was prepared by utilizing 5 mg/mL of the concentration. A total of 2.3 mL of HBSS needs to be added to prepare the master stock and later needs to be divided into aliquots. In an individual experiment, 168 μL of liberase from the aliquot was added into 10 mL of HBSS. The samples were incubated using 5 mg/mL liberase in HBSS for 15 min with rocking in a 37 °C incubator, 4.5% CO_2_. The colon crypts were collected by scraping the colons (cells off from the basement membrane by using a pipette tip and forceps). The crypts isolated were passed through a 100 µm cell sieve. The cells were then subjected to a gentle spin at 3000× *g*, 4 °C for 5 min. The gradient assay was employed. The goblet cell pellet was re-suspended in freezing medium (6:3:1) Medium: Fetal bovine serum (FBS): DMEM and was transferred into a cryo-vial and stored for future use.

### 2.4. RNA Extraction and cDNA Conversion

The RNeasy Mini kit was employed (Qiagen, VIC, AUS) with RNase-Free DNase set (Qiagen, AUS) to extract RNA from LS174T cells (3 × 10^5^ cell population). The RNA quantitative and qualitative analysis was performed by using an Experion automated electrophoresis system (Bio-Rad Laboratories, VIC, AUS) and RNA samples with an RNA quality index (RQI) >7.0 were considered suitable for expression analysis. The iScript cDNA synthesis kit (Bio-Rad Laboratories, AUS) was used to transcribe one microgram of total RNA to cDNA as per the manufacturing protocol.

### 2.5. qRTPCR

The RT-PCR was performed by using Taqman^®^ probes (Life Technilogies, AUS) for *GAPDH* (Hs03929097_gl), *ATF6* (Hs00232586_m1), *XBP1* (Hs00231936_m1), *GRP78* (Hs0060719_gH), *CHOP* (Hs00358796_g1), and *PERK* (Hs00984006_m1). The RT-reaction mixture consisted of 40 ng of cDNA, Taqman Fast Advanced Master mix (Life Technologies, AUS), and 1 µL of gene-specific probe/total volume of 20 µL. The reactions were run in duplicates on the qRT-PCR machine (StepOne Plus-Life Technologies, VIC, AUS). Thermo cycling conditions included 90 °C for 20 s, 40 cycles at 95 °C for 1 s, and 60 °C for 20 s. Gene expression was quantified by using the comparative (ΔΔCT) method where the threshold cycle (CT) for each gene was normalized to reference gene *GAPDH*.

### 2.6. The Assessment of Cytokines by Bioplex

Ls174T cells were utilized for measurements of IL-8, IL-4, IL-6, and IL-10 cytokine levels. The cells were subjected to the corresponding treatments of tunicamycin, 4-phenylbutyric acid (4PBA) and YM155. The cells were cultured in 12-well cell culture plates (Greiner, VIC, AUS) and were seeded at a density of 3.0 × 10^5^ cells for every well in 2.0 mL of medium and incubated overnight at 37 °C/5% CO_2_ to enable the cells to adhere. Medium was replaced the following day comprising of the treatments: Tunicamycin (10 µg/mL in DMSO) and 4PBA (40 µg/mL) incubated for 6 h at 37 °C/5% CO_2_. After 6 h of treatment, the media from each well was collected and utilized for measuring cytokines by utilizing the Bio-Plex^®^ Pro human cytokine assay kit (Bio-Rad^®^), which is indicated by the manufacturer protocol. Additionally, 50 µL of cytokine beads were added to the 96-well plate and incubated for 30 min before washing twice with wash buffer. At that point, 50 µL of every standard including blank tests were added to the individual wells and incubated at room temperature on a shaker at 850 rpm for 30 min. Post incubation, the wells were washed thrice and 25 µL of detection antibody was added to each well and incubated at room temperature on a shaker at 850 rpm for 30 min. Subsequently, 50 µL of streptavidin-PE was added to each well and incubated at room temperature in a shaker at 850 rpm for 10 min. After three washes, 125 μL assay buffer was added to each well and incubated at room temperature for 30 s. After incubating, the plates were read on the Bio-Plex^®^ 200 system and analyzed by using in Bio-Plex Data ProTM Software (Gladesville, NSW, AUS). All the tests were performed in triplicate.

### 2.7. Toxicity Assay Lactate Dehydrogenase (LDH)

After incubation with the respective treatments, the supernatants were collected for the determination of cytotoxicity by using the lactate dehydrogenase (LDH) assay. The cellular cytotoxicity was assessed by the LDH in-vitro cytotoxicity assay (TOX7, Sigma-Aldrich, St. Louis, MO, USA). The culture supernatants were centrifuged at 250× *g* for 4 min. An aliquot containing 50 µL of either blank (complete medium) or control (cells only) and cells treated with tunicamycin (TUN) supernatants obtained after the respective time point incubations was mixed with 100 µL of a solution containing the LDH assay mixture (LDH substrate, LDH dye, and LDH cofactor). The mixture was then incubated at room temperature for 20 to 30 min and the reaction was quenched by the addition of 1*N* hydrochloric acid (15 μL). The absorbance was measured spectro-photo-metrically by using a plate reader (Spectra Max M2 microplate reader, Sunnyvale, CA, USA) at a wavelength of 490 nm. The cellular viability was examined by a Trypan Blue exclusion staining assay using a Countess Automated Cell Counter (Thermo-Fisher, Waltham, MA, USA).

### 2.8. Western Blot

The cells (1 × 10^6^ treated for 6 h) post treatments were washed with HBSS followed by homogenization in 2 mL of RIPA buffer/10% of Protease Inhibitor (Sigma-Aldrich, AUS). After subjecting to centrifugation at 12,000 rpm for 20 min at 4 °C, the supernatant was collected. Thirty micrograms of protein from each sample was denatured in Laemmli loading buffer (Bio-Rad Laboratories, AUS) and separated on precast 12% SDS-PAGE gels (Bio-Rad Laboratories, AUS), which was followed by overnight transfer onto Polyvinylidene difluoride (PVDF) membranes (Millipore, NSW, AUS) at 30 mV at 4 °C. By using 5% non-fat milk, the blot was blocked before incubating with respective antibodies: anti-GADPH, Survivin, *Grp78* (#14C10, 1:3000, Novus Biologicals, AUS), poly(ADP-ribose)polymerase (PARP), and cleaved PARP (SIGMA, VIC, AUS) overnight at 4 °C in blocking buffer. The blot was washed in PBST and incubated with appropriate species monoclonal horseradish peroxidase-conjugated anti-IgG secondary antibodies (1:5000) for 1 h at 20 °C. The bands were visualized by using the Supersignal West Pico chemi-luminescent kit (Thermo Scientific, VIC, AUS). It was digitized and the band intensities were determined by using a Fuji LAS-3000 Imager (Fuji Life Sciences, JPN). The samples from all groups were included in each individual blot to ensure accurate quantification across multiple blots.

### 2.9. Apoptosis Assays

#### 2.9.1. Caspase-3 Assay

The Caspase-3 fluorimetric test was performed on the cell lysates, which were collected after the 6-h treatment as indicated by the guidelines, which gave the test unit and past reference. This compound test depended on the hydrolysis of the caspase-3 peptide substrate (acetyl-Asp-Glu-Val-Asp or AC-DEVD) conjugated to a fluorochrome at the C-terminal Asp, which brings the arrival of the fluorescent moiety. After the fluorimetric treatment, the fluorescence (absolute units) was estimated by utilizing the CytoFluor Multi-Well Plate Reader Series 4000 spectro-fluorometer from PerSeptive Biosystems (Framingham, MA, USA) as per the manufacturer’s protocol.

#### 2.9.2. Annexin V Assay

The Annexin-V-Fluos assay (cat. No. 11828681001, Roche Diagnostics, Zug Switzerland) was utilized to measure apoptotic (annexin V) cell populations. The cells after the 6 h treatments were incubated in incubation buffer (10 mM HEPES at pH 7.4, 140 mM NaCL, 2.5 mM CaCl_2_) and supplemented with annexin V–PI mix for 15min atroom temperature (RT) and the cells were analyzed by flow cytometry and confocal microscopy with 4′,6-diamidino-2-phenylindole(DAPI) as nuclear staining and the Fluorescein isothiocyanate (FITC) channel (fluorescein) for visualizing apoptosis cells (Nikon AR1MP) per the manufacturers’ protocol.

### 2.10. Proliferation Assay

The proliferation test 5-ethynyl-2′-deoxyuridine (EdU) (Invitrogen, VIC, Aus) was executed according to the guidelines included by the producer, which means the upgraded convention. The Click-iT™ EdUIF Assay Kit, Invitrogen™ was included at a 50 μM final concentration. After the respective treatments, the cells were subjected to 3 × 5 min PBS washes. A cell volume of 20–30 μL was directly incubated at room temperature (RT) in the EdU detection cocktail (Invitrogen, Click-iTEdU Alexa Fluor 488 HCS assay, *cat no: {“type”:“entrez-nucleotide”,“attrs”: {“text”:“A10027”,“term_id”:“492344”,“term_text”:“A10027”}}* *A10027*) for 30 min. For a one sample reaction, the following amounts of the kit components are mixed in 144 μL distilled water: 1.6 μL buffer additive (component F, kept frozen in small aliquots), 14 μL reaction buffer (Component D), 6.7 μL Copper (II) sulfate solution (Component E, 100 mM CuSO_4_), and 0.07 μL Alexa Fluor 488 azide (Component B, in 70 μL DMSO). Confocal laser scanning microscopy was performed by using the Olympus Fluoview FV1000 confocal laser scanning microscope (Olympus Life Science Europa GmbH, Hamburg, Germany). Alexa Fluor 488 images were captured under excitation: 460–490 nm, dichroic beam splitter 505 nm, and emission of 510–550 nm. The images were analyzed by using ImageJ software (Madison, WI, USA).

### 2.11. Statistical Analysis

Statistical significance of the differences between groups among repeated experiments was calculated by one-way and two-way ANOVA and the Fisher’s LSD-test using GraphPad Prism 7 software (GraphPad Software Ltd., La Jolla, CA, USA). The results are expressed as the mean values ± standard deviation. In all statistical tests, a *p*-value < 0.05 was considered statistically significant.

## 3. Results

### 3.1. Increased Survivin Expression in the Colon of Winnie

**Figure 1:**
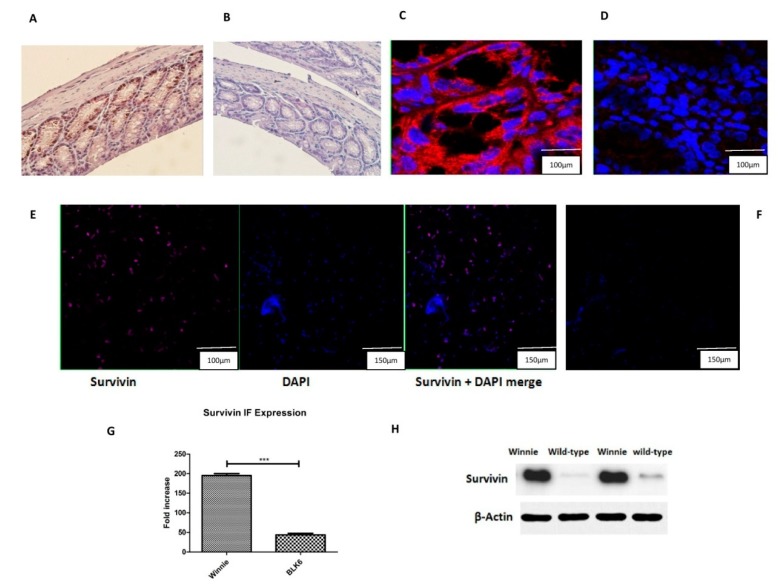
Expression of Survivin in Winnie. (**A**) Expression of Survivin in Winnie colon tissue. (**B**) Expression of Winnie in control black 6 (Blk6) colon tissue. (**C**) Double staining of Survivin in *Winnie* colon (Red-Survivin, Blue-4′,6-diamidino-2-phenylindole (DAPI)). (**D**) Double staining of Survivin in Blk6 colon (Red-Survivin, Blue-DAPI). (**E**) Representation of double staining of Survivin on colonic goblet cells of Winnie (violet- Survivin, blue-DAPI). (**F**) Representation of double staining of Survivin on colonic goblet cells of Blk6 (violet- Survivin, blue-DAPI). (**G**) Immunofluorescence (IF) quantification of Survivin expression in goblet cells. *** represents *p* < 0.001. (**H**) Protein expression by Western blot for Survivin in the Winnie and Blk6 colonic goblet cells standardized by β-Actin.

We utilized the *Winnie* mouse model [13] of chronic ERS and control black 6 (Blk6) mice to assess the expression of Survivin in the colon since there was an increased ERS documented in the Winnie goblet cells. We have first checked the expression of Survivin in the colon tissue by immunohistochemistry (IHC) and immunofluorescence (IF). IHC revealed an increase in the expression of Survivin in the colonic epithelial cells, which is shown in Figure 1A,B. Similar to that of the IF where there was a striking elevated expression observed in Winnie compared to that of wild type mice where there was hardly any expression of Survivin (Figure 1C,D). We further isolated the goblet cells from the colons of both Winnie and Blk6 mice and performed IF analysis to precisely define the Survivin expression in the cells. There was a significant increase in the expression of Survivin in Winnie goblet cells compared to the wild type Blk6 (Figure 1E,F). We have also evaluated the Survivin protein expression of the goblet cells by using a Western blot where the Survivin protein expression of the goblet cells was found to increase in Winnie compared to the wild type mice (Figure 1H).

To further assess the functional relationship between Survivin and ERS, we employed an in vitro evaluation on a similar goblet cell like mucous secretory epithelial cell line (LS174T) to determine the functional and downstream effects of ERS induction and inhibition in the context of cancer and its emerging effects.

### 3.2. Dynamics of Survivin in Line with Endoplasmic Reticular Stress (ERS)

**Figure 2:**
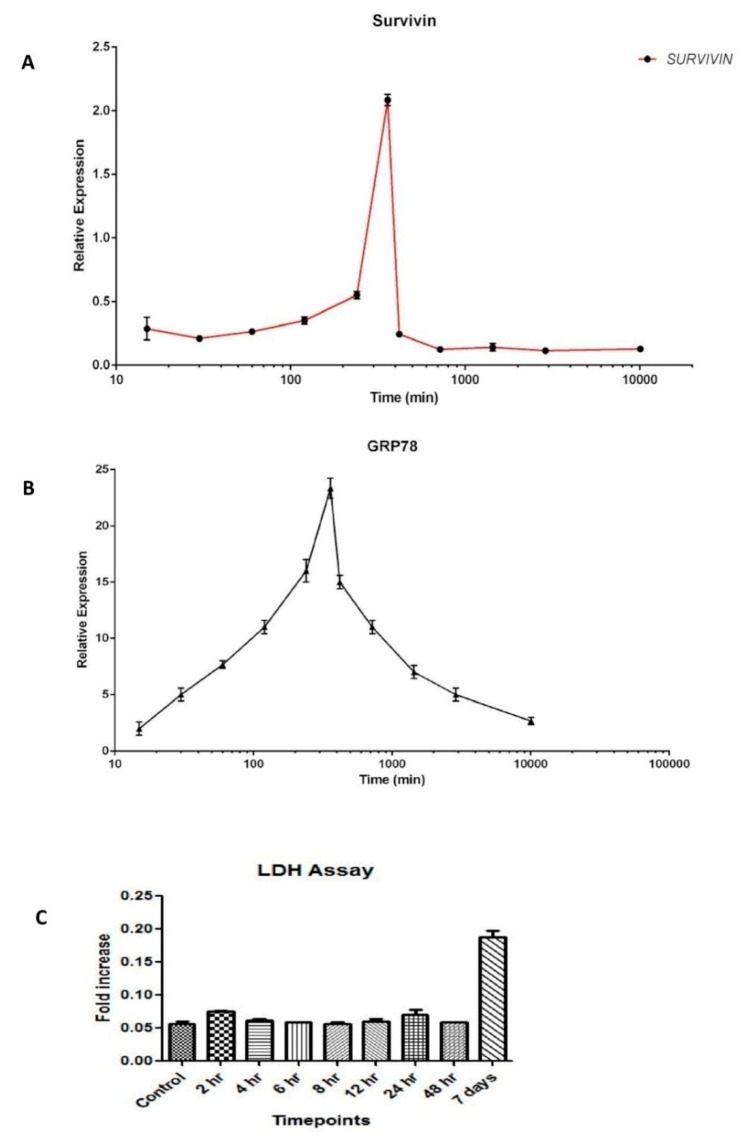
Molecular dynamics of Survivin with respect to Endoplasmic Reticular Stress (ERS). (**A**) Molecular dynamics of Survivin in the LS174T cell line with ERS treatment by TUN 10 µg/mL. (**B**) mRNA dynamics release of *Grp78* in the LS174T cell line with ERS treatment by TUN 10 µg/mL. (**C**) LDH assay of the LS174T cells with ERS treatment by TUN 10 µg/mL.

Having determined the expression of Survivin in Winnie goblet cells, we then investigated the dynamics in LS174T cells. First, to study the molecular dynamics of Survivin in parallel with ERS, we treated LS174T cells with TUN over a period of seven days and collected RNA at various time points (0 min, 15 min, 30 min, 1 h, 2 h, 4 h, 6 h, 8 h, 12 h, 24 h, 48 h, 72 h, and 7th day) to determine mRNA expression. *Grp78* was used as a standard marker of ERS. The lactate dehydrogenase assay (LDH) was also performed at various time points to monitor the extent of cell death. It was evident from quantitative real-time PCR that the expression of Survivin was greatest (2.5-fold increase vs. control) at 6 h (360 min) of TUN treatment and this increase in Survivin expression was concomitant with a similar increase in the ERS maker *Grp78* (Figure 2A,B). The expression pattern of surviving showed a steady increase and reached the highest level by the sixth hour post TUN treatment. After the sixth hour time point, there was a steady drop in Survivin expression resulting in no expression by the 900th minute (Figure 2A). Similar to Survivin expression, ERS induction by TUN showed an expected pattern of *Grp78*.

ERS-mediated cell death was shown through the LDH assay. There was no cell death recorded in the LDH assay during the sixth hour time point, which also proves that there was no death occurring during high ERS under TUN treatment.

### 3.3. Inhibition of Endoplasmic Reticular (ER) Stress Is Correlated to Survivin Expression in the LS174T Cell Line

**Figure 3:**
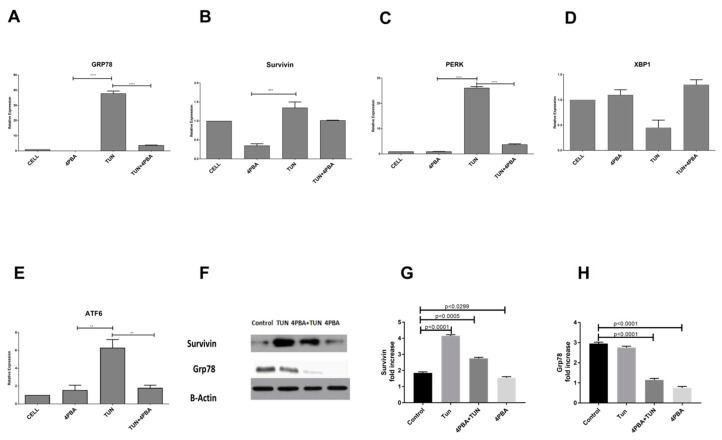
Expression of *UPR* genes and Survivin modulated by ERS induction and inhibition. mRNA expression of *UPR* genes: (**A**) *Grp78*, (**B**) Survivin, (**C**) *PERK*, (**D**) *XBP1*, and (**E**) *ATF6* in LS174T cells treated with TUN 10 µg/mL and 10 mM of 4PBA for 6hours normalized to *GAPDH* (*n* = 3). Data are shown as the mean fold change  ±  SEM (significance testing vs. TUN: **, ***, ****; *p* < 0.01, *p* < 0.001 and *p* < 0.0001, respectively). (**F**) Representative Western blot for Survivin and *Grp78*. (**G**) Western blot quantification of Survivin. The bands were normalized with β-Actin and quantified. (**H**) Quantification of Western blot of *Grp78*.

Having established the dynamics of Survivin expression under ERS conditioning, we further aimed to establish the relationship between Survivin, ERS, and the inhibition of apoptosis. Accordingly, the LS174T cells were incubated with an ERS inducer TUN (10 µg/mL) for 6 h since it was the time point in the previous assay with the highest ERS and the *UPR* genes (*Grp78*, *XBP1*, *PERK*, and *ATF6*) were assessed. We observed an increase expression of *UPR* genes concomitant with an increase in Survivin expression. Upon treating with TUN, the expression of GRP78 rose to 35-fold, which was diminished under ERS inhibition by 4PBA (Figure 3A). The expression of *PERK* was dropped to two-fold in a 4PBA treated group compared to the TUN group, which was 25-fold increased (Figure 3C). A similar significant drop of UPR genes was observed in *ATF6* where the expression was declined to two-fold in the 4PBA group in comparison to the TUN treated group, which was six-fold (Figure 3E). A significant expression of Survivin was reduced under ERS inhibition where the relative expression reduced to 1.25-fold from 0.31 (Figure 3B). There was no significant expression of XBP1 recorded in our study upon ERS induction by TUN. This was similar to the expression of IRE1 (Figure S1) which could be because of the regulation of XBP1 by IRE1 (Figure S2). We have then performed the Western blot analysis of the protein extract from the LS174T cells treated with TUN and 4PBA. There was a noticeable significant increase in protein expressions of both Survivin and *Grp78* in the groups induced with ERS and a significant reduction in expressions of *Grp78* and Survivin observed in groups under reduced ERS by 4PBA (Figure 3F–H). These results depict the relationship of ERS and the inhibition of apoptosis.

**Figure 4:**
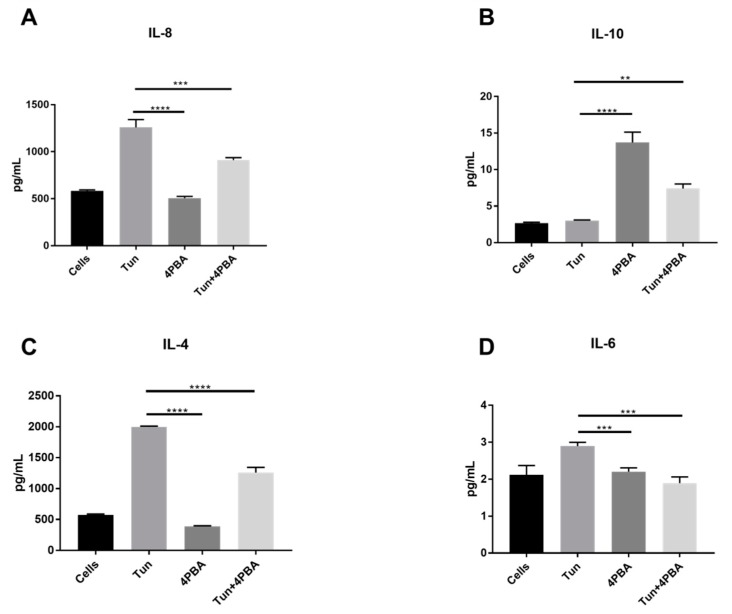
Cytokine expression of (**A**) IL8, (**B**) IL10, (**C**) IL4, and (**D**) IL6 in LS174T cells treated with TUN 10 µg/mL TUN and 10 mM of 4PBA. (significance testing vs. TUN: **, ***, ****; *p* < 0.01, *p* < 0.001 and *p* < 0.0001, respectively).

Since the ERS inhibition could reduce UPR markers and inhibition of apoptosis, we wanted to evaluate the effects of ERS reduction in ameliorating ERS mediated inflammation (Figure 4). 4PBA successfully modulated pro-inflammatory and anti-inflammatory cytokines in our study. TUN at a dose of 10 µg/mL could successfully induce ERS-mediated inflammation in LS174T cells evident through the up regulation of pro-inflammatory cytokines IL8, IL4, and IL6 from 584 ± 9.1, 573.52 ± 15.84, and 2.11 ± 0.19 pg/mL to 1258.9 ± 59, 1996.84 ± 3.6, and 2.9 ± 0.1 pg/mL, respectively (*p* < 0.00001) (Figure 4). ERS inhibition by 4PBA significantly reduced the inflammatory cytokines to 507.1 ± 16.3, 388.57 ± 10.61, and 2.2 ± 0.1 pg/mL, which offers a strong protection from inflammation (Figure 4). The 4PBA and TUN group also offered significant protection against inflammation at 910.2 ± 30.12, 1258.39 ± 89.6, and 1.89 ± 0.11 pg/mL (*p* < 0.001). The anti-inflammatory cytokine IL10 was also substantially modulated by inhibiting ERS. Upon inflammation, IL10 was standing at 3.0 ± 0.1 pg/mL and, after co-treatment with 4PBA, the value of IL10 rose to 13.7 ± 1.1 pg/mL alongside the combined group (4PBA and TUN) with a surprising 7.4 ± 0.6 pg/mL, which signifies that not only does ERS inhibition lead to reduction in pro-inflammatory cytokines but also regulates anti-inflammatory cytokines.

### 3.4. Inhibition of ERS Escalates Apoptosis in Colon Cancer Cells

**Figure 5:**
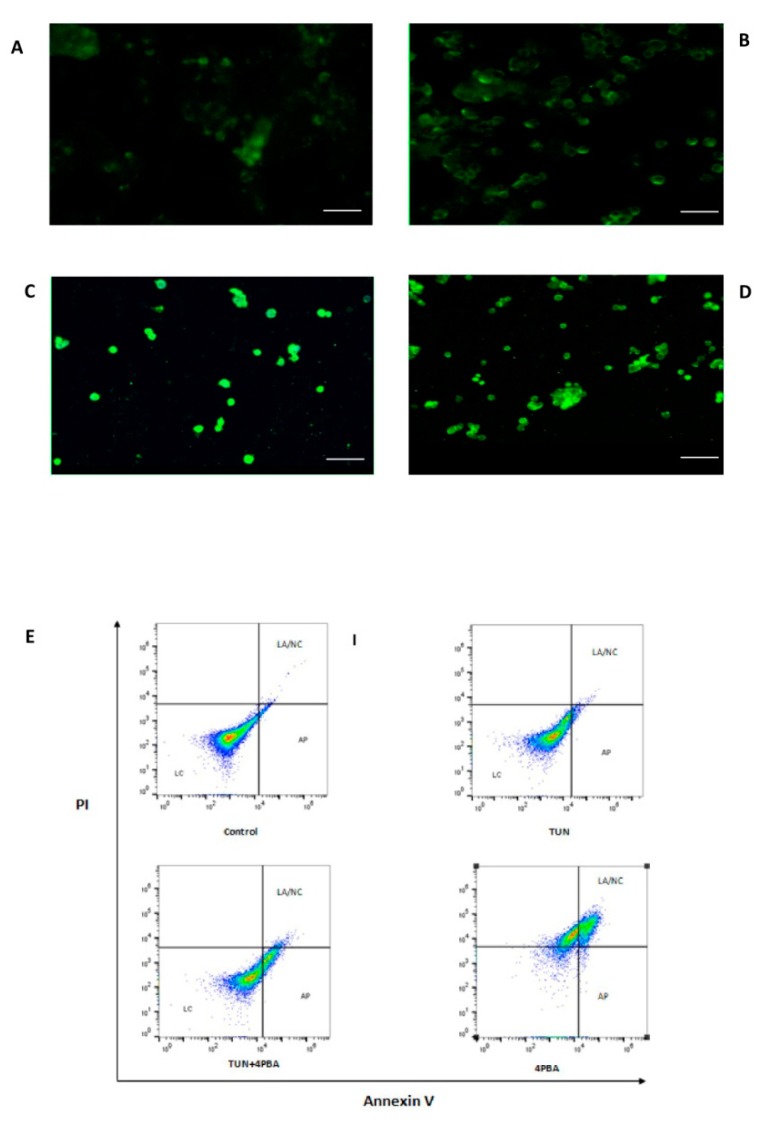
The effect of ERS on apoptosis by the Annexin V assay. The Annexin V assay of LS174T cells treated with 10 µg/mL TUN and 10 mM of 4PBA for 6 h stained with Annexin V-FITC and analyzed by confocal microscopy. (**A**) Cells not subjected to any treatment (control). (**B**) Cells treated with TUN alone. (**C**) Cells treated with TUN + 4PBA. (**D**) Cells treated with 4PBA alone. The graphs represent the flow cytometry analysis after respective treatments. (**E**) Control group containing non-treated cells that were viable and not undergoing apoptosis. Cells treated with TUN and not prone to apoptosis. Cells treated with TUN + 4PBA, which have started shifting towards the apoptotic window (early apoptosis). Cells treated with 4PBA and in the end-stage apoptosis or already dead. Additionally, there was an increase in dead cell populations observed. (The axis of the flow cytometry is represented by PI (Propinium iodide and Annexin V. LA represents late apoptosis. NC represents necrotic. LC represents live cells and AP represents apoptotic). Scale bar represents 75 µm.

To validate the induction of apoptosis with relation to TUN mediated effects, LS174T cells were subjected to ERS induction with 10 µg/mL TUN and ERS inhibition by 10 mM of 4PBA and apoptosis was quantified by staining with Annexin V (FITC). There was an increase of apoptosis in the cells treated with 4PBA alone when compared to TUN and cells alone groups. Cells treated with 4PBA alone also demonstrated a change in their morphology such as a disoriented nucleus when compared to the control group and TUN group (Figure 5D). There was a significant increased apoptosis recorded through the FITC flow cytometry where the apoptotic cells were plotted through the graph represented by the 4PBA group. There was a shift of cells from the live to the apoptotic column (Figure 5E) observed in the flow cytometry graph compared to the TUN where the live cell numbers were denoting a higher increased live cell number and higher control groups where the live cells were geared towards live and pre-apoptotic systems (Figure 5E). The cells were shifting to early apoptotic in the TUN+4PBA group and towards necrotic or late apoptotic systems in the 4PBA alone group.

**Figure 6:**
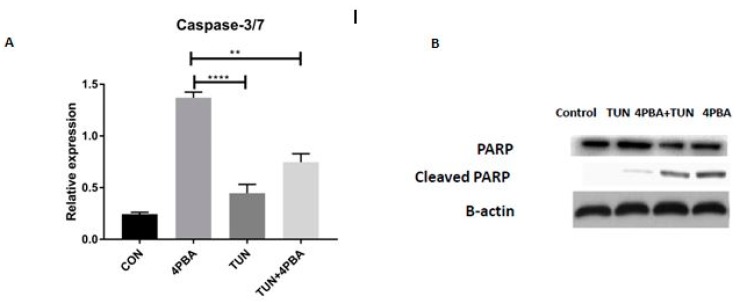
Influence of ERS on Caspase3/7 and PARP cleavage assays. (**A**) Effect of Caspase3/7 assay on LS174T cells (control or non-treated, 4PBA at a concentration of 10 mM, TUN at a concentration of 10 µg/mL and TUN + 4PBA). (**B**) Western blot of PARP and cleaved PARP of respective groups normalized with β-Actin. (significance testing vs. TUN: *** *p* < 0.001 and **** *p* < 0.0001 respectively).

To investigate the occurrence of programmed cell death due to Caspase activation, cell supernatants from the treated LS174T cells were subjected to the Caspase 3/7 assay. In this case, we have observed a significantly increased Caspase 3/7 expression in the 4PBA alone group compared to the 4PBA + TUN and TUN groups (Figure 6A). Parallel to this, the cleavage of PARP, which is an executioner Caspase substrate, was also detected by using Western blot analysis. The cleaved PARP (Figure 6B) was increased in the group treated with 4PBA (ERS inhibition), which suggests that 4PBA increases apoptosis partially through mitochondrial-mediated cell death and also that reducing the protein folding by 4PBA leads to the initiation of cell death.

### 3.5. ERS Exhibits Direct Relationship to Proliferation in the LS174T Cell Line

**Figure 7:**
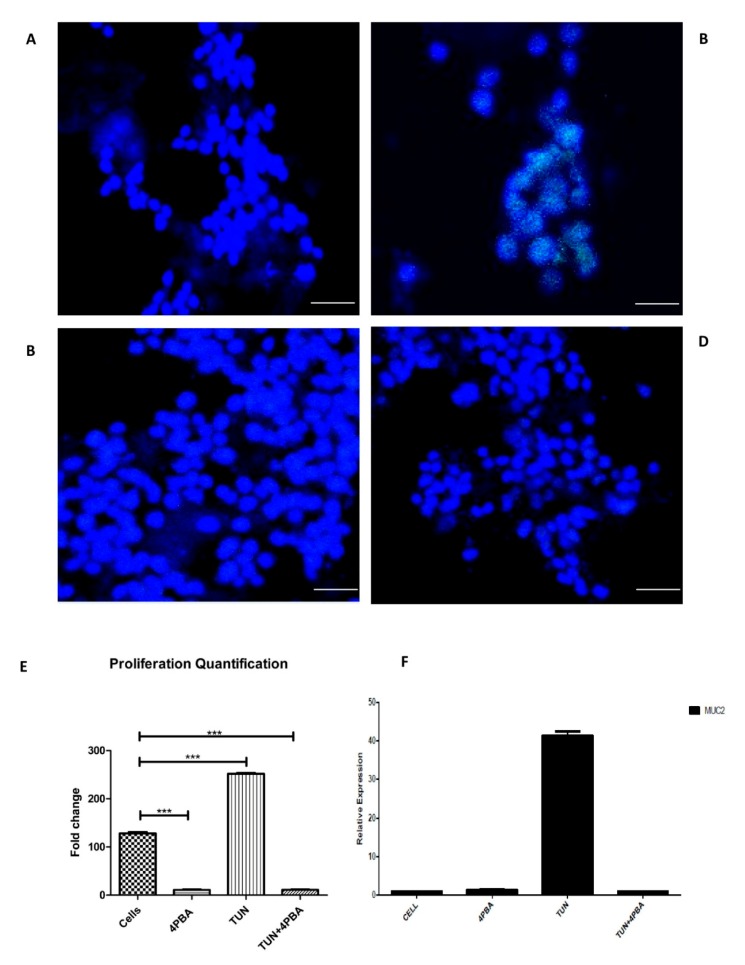
The effect of ERS on the proliferation rate and *Muc2* expression in LS174T cells. Cell proliferation assay performed by a 5-ethynyl-2′-deoxyuridine (EdU) click assay after incubating with treatments. (**A**) The cells alone or non-treated (**B**) TUN at a concentration of 10 µg/mL. (**C**) 4PBA at a dose of 10 mM. (**D**) TUN + 4PBA. (**E**) Quantification of the proliferation assay by Image J^®^ representing different groups. (**F**) *Muc2* gene expression (cells after treatments were subjected to RT-PCR). Scale bar represents 50 µm. (significance testing vs. TUN: *** *p* < 0.001 respectively).

After evaluating the process of ERS inhibition, which leads to an increase in apoptosis, we decided to determine the cellular proliferation with regard to ERS inhibition and induction. The 5-ethynyl-2′-deoxyuridine EdU assay for in-vitro proliferation was employed for this purpose. Following ERS induction and inhibition, the LS174T cells were seeded in eight chamber wells and incubated with an EdU click assay reagent and visualized through confocal microscopy. Images were quantified by Image J^®^ software. We observed a reduction in cellular proliferation observed in 4PBA groups when compared to the TUN group where the proliferation was significantly increased (Figure 7B) and when compared to the control group. Proliferation increased by TUN was almost 250-fold compared to the ERS inhibition groups (4PBA alone and 4PBA + TUN) where the proliferation rate dropped to 10-fold from 250 (Figure 7E).

We further correlated *Muc2* gene expression with a proliferation rate under ERS. We performed RT-PCR for *Muc2* expression with ERS induction and inhibition groups (Figure 7F). The data revealed the increased expression of *Muc2* with the induction of ERS by 10 µg/mL TUN to 40-fold, which denotes the relation of ERS and proliferation. ERS inhibition by 4PBA at a dose of 10 mM reduced the *Muc2* expression significantly five-fold. A similar expression was noted in the TUN + 4PBA group.

### 3.6. YM155, a Survivin Inhibitor, Subsides ERS and ERS Mediated Inflammation

**Figure 8:**
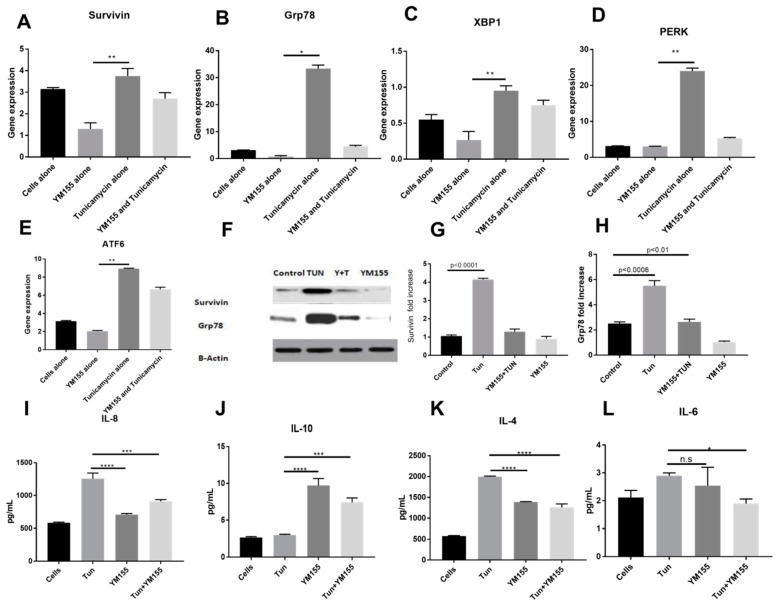
Gene expressions of *UPR* genes, Survivin, and measurement of inflammatory cytokines of cells treated with YM155.mRNA expression of *UPR* genes: (**A**) IAP gene Survivin, (**B**) *Grp78*, (**C**) *XBP1*, (**D**) *PERK*, and (**E**) *ATF6* in LS174T cells treated with TUN 10 µg/mL and 100 nM of YM155 for 6hours normalized to *GAPDH* (*n* = 3).The data are shown as the mean fold change ± SEM (significance testing vs. TUN: *, **, ***, ****; *p* < 0.05, *p* < 0.01, *p* < 0.001 and *p* < 0.0001, respectively). (**F**) Representative Western blot for Survivin and *Grp78*. The bands were normalized with β-Actin and quantified. (**G**,**H**) represents Western blot quantification. Cytokine expression of (**I**) IL8, (**J**) IL10, (**K**) IL4, and (**L**) IL6 in LS174T cells treated with TUN 10 µg/mL and 100 nM of YM155.

Since we could inhibit Survivin expression and inflammation under reduced ERS condition, we wanted to investigate whether we could show the converse effect of Survivin inhibition and its effect on ERS. Consistent with the ERS inhibition, Survivin inhibition by YM155 also resulted in the decrement of ERS markers both at the transcription and the protein level. We investigated the potential of Survivin inhibition in ameliorating ERS in the LS174T cell line. Exposure to TUN 10 µg/mL resulted in a significant increase of the UPR genes along with Survivin. Upon treatment with YM155, the expression of Survivin as expected reduced from 3.2 fold to 1 fold (shown in Figure 8A). YM155 showed marked reduction of UPR genes like *GRP78* from 30-fold to two-fold (Figure 8B). Additionally, 0.9-fold to 0.2-fold for *XBP1* (Figure 8C) and a startling four-fold from 22-fold for *PERK* (Figure 8D) and a drop to 1.8-fold from eight-fold for *ATF6* (Figure 8E).

Similar responses were also observed in the cellular protein levels of *Grp78* and Survivin as shown by Western blot analysis where Survivin inhibition also lead to ERS inhibition (Figure 8F–H), which confirms that modulation of mRNA expression is reflected in active protein suppression of Survivin and ERS through *Grp78* as well.

Functional inhibition of Survivin showed a striking change in inflammation in LS174T cells where YM155 modulated IL8, IL4, and IL6 (Figure 8I,J,L). The grounded levels of cytokines (584.48 ± 9.7, 573.5 ± 15.8, and 2.11 ± 0.19 pg/mL) increased to 1258.39 ± 89.6, 1996.8 ± 13.42, and 2.9 ± 0.1 pg/mL. Upon treatment with YM155, there was a marked decrease in pro-inflammatory markers to 707.18 ± 16.2, 1388.8 ± 10.38, and N.S pg/mL (*p* < 0.0001). There was also a positive variation in the anti-inflammatory cytokine IL10 from 3.0 ± 0.1 to 9.7 ± 1.1 upon YM155 treatment shown in Figure 8J. Combined treatment of YM155 with TUN also exhibited significant activity where the cytokine levels were 910.27 ± 29.8, 1258.30 ± 13.10, and 1.89 ± 0.1 pg/mL (*p* < 0.0001).

### 3.7. YM155 Initiates Cell Death in LS174T Cells via Caspase3/7

**Figure 9:**
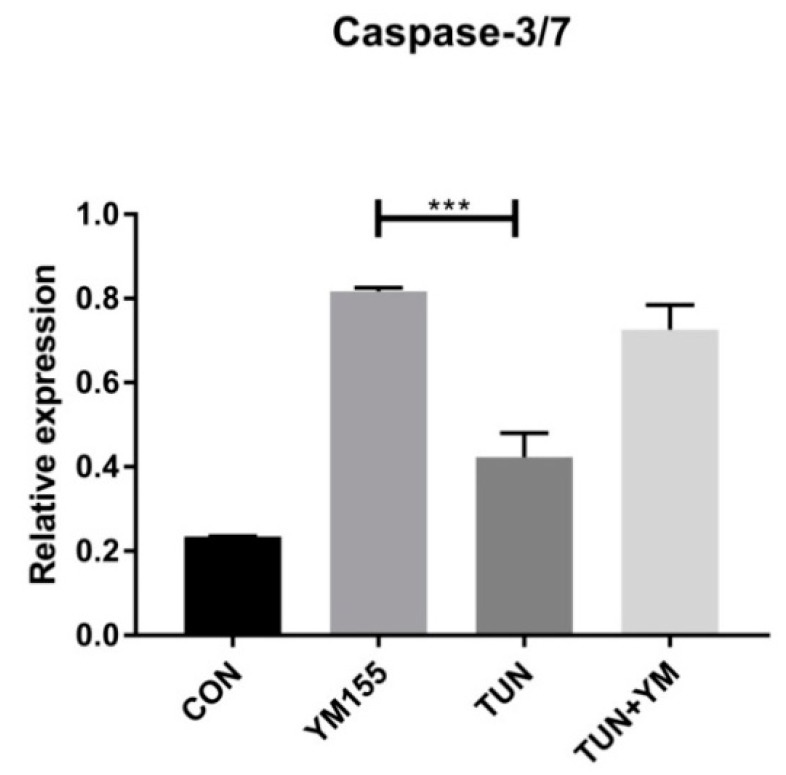
The effect of Survivin inhibition on Caspase 3/7 assay. LS174T cells were treated with YM155 for 6 h and apoptosis was determined by the Caspase 3/7 assay representing the following groups:control or non-treated, YM155 at a concentration of 100 nM, TUN at a concentration of 10 µg/mL, and TUN + YM155. *** represents *p* < 0.001, respectively.

As IAPs target the Caspase pathways specifically, we wanted to evaluate whether Survivin inhibition by YM155 could elevate Caspase 3/7. Figure 9 presents the effect of YM155, which, at a concentration of 100 nM, increased the expression of Caspase 3/7 significantly when compared to the TUN group and also the control group. The YM155 + TUN group also induced Caspase 3/7 activity. This clearly confirms that Survivin inhibition may lead to cancer cell apoptosis in the intrinsic death pathway.

## 4. Discussion

Our study provides evidence that ERS mediated signaling pathways are one of the major protective mechanisms adopted by cancer cells where they inhibit the ERS responses including apoptosis. We have described the role of Survivin in the above described mechanism. In the present study, we have demonstrated the effects of ERS inhibition by 4PBA over reduction of UPR markers and also Survivin. We have also enumerated that ERS inhibition leads to amelioration of ERS mediated inflammation, increases apoptosis in LS174T cells, and lessens cancer cell proliferation. First, in order to validate the expression of Survivin in vivo, we determined the expression of Survivin in the colon tissue and goblet cells isolated from Winnie, which is a chronic model of severe ERS. We noticed that Survivin expression was elevated compared to the wild type, which presents a similar trend to that of previous studies on colon cancer [16,19]. This prompted us to further investigate goblet cell like *Muc2* expressing the cell line LS174T for linking between Survivin and ERS. Even thoughJayakumar et al. has explored the molecular dynamics of Survivin recently in cancer cells [20], the expression dynamics was not described with regard to ERS induced by chemical agents such as TUN. The 6th hour time point, which was regarded as the highest expression of Survivin, along with *Grp78* identified this as the end time point for all the experiments in our studies into the path of ERS-mediated apoptosis.

It is well established that ERS leads to pathogenesis of numerous diseases including cancer. The ERS aids in the adaptation against non-favorable conditions and is involved in tumor growth. The UPR markers namely IRE1, *XBP1*, *PERK*, and *Grp78* are involved in tumorigenesis [21]. ER resident chaperones including calreticulin lowered cell death and have been associated with ERS induction in tumor cells [21]. TUN at a dose of 10µg was able to increase the expression of *UPR* genes (*Grp78*, *PERK*, *XBP1*, and *ATF6*) and IAP protein Survivin, which indicates the role of ERS in tumor promotion. Likewise, 4PBA reduced the expression of all ERS markers (*UPR* genes) alongside the expression of Survivin, which denotes that ERS inhibition leads to Survivin reduction. We have additionally evaluated the potential of ERS inhibitor 4PBA in ameliorating the ERS mediated inflammation induced by TUN. In recent studies, 4PBA was employed to ameliorate LPS-induced lung inflammation through modulation of the NF-κB/IκB and HIF-1αsignaling pathways, which highlights the benefit of reducing ERS [22].

There is a mounting evidence involving cytokines such as IL8, IL4, and IL6 in inflammation mediated carcinogenesis. IL8 expression correlates with the angiogenesis, tumorigenicity, and metastasis of tumors in numerous xenograft and orthotopic in vivo models [23]. IL8 in association with CXCR1 was related to a high prognosis of human pancreatic cancer [24]. 4PBA exhibitedareduction of IL-8 significantly when compared to the TUN group in our study and suggests that ERS inhibition may lead to reduced tumorigenesis. As stated earlier, interleukins are involved in growth, differentiation, and migration in various cancers. Apart from decreasing apoptosis in cell lines such as HCT116 [25], IL4Rα contributed to tumor formation in a mouse model of colitis-associated cancer and mediated proliferation of epithelial tumor cells [25]. Previous studies indicated efficacious treatment against cancer due to the blockade of IL4 where the functional role of IL4 cytokine was demonstrated as an important mechanism that protects the tumorigenic CD133+ cells from apoptosis [26]. Similar to the above mentioned description, our data also demonstrated a role of IL4 in TUN mediated inflammation. We aimed to observe the effects of ERS inhibition by 4PBA over IL4 in TUN-mediated inflammation where we observed a significant reduction of IL4 levels in the groups (4PBA and 4PBA + TUN), which was in line with previous findings where the blocking of IL4 signaling sensitized cancer stem cells (CSCs) to apoptotic stimuli and increased the in vivo efficacy of cytotoxic therapy [27]. Additionally, in colon cancer stem cells, Survivin was regulated by IL4 through STAT-6 signaling and allowscancer cells to escape chemo sensitizing [28], which can be explained through our study that ERS inhibition may have reduced Survivin expression via IL4. The fact that the ERS inhibition in our study with 4PBA led to IL-10 elevation was a significant finding because IL10 stimulated cytotoxicity in CD8+ cells and cancer cells treated with pegylated IL-10 led to tumor rejection, anti-tumor effects, and tumor-associated inflammation [29]. It was also notable that IL10^−/−^ mice were found to be particularly susceptible to chemically induced skin cancers, which highlights its protective role. IL10 along with PD-1 exhibited an anti-tumor response in ID8 ovarian tumor bearing mice, which leads to a decrease in tumor mass and a significant increase in survival [30]. Lastly, IL6 evaluated in our study was previously related to an advanced stage of CRC development [31] where it was proved that IL6 promoted tumor cell proliferation and inhibited apoptosis through gp130 activation. IL6 levels not only increased in patients but also in the tumor. The same study reported that IL-6 levels greatly determined patient survival. IL6 was one of the cytokines, which was linked directly between IBD and CRC [31]. 4PBA in our study reduced the expression of IL-6 along with other inflammatory cytokines, which can be related to previous studies of attenuation of angiogenesis and tumor formation in colon cancer [32].

UPR mediators are regarded as being fundamental to ERS-mediated apoptosis [33]. Knocking down of *Grp78* in tumor-associated endothelial cells led to an increase in apoptosis, which reveals that ER chaperone inhibition would result in reducing carcinogenesis [34]. The evaluation of early apoptosis was carried out by Annexin V flowcytometry and IF assays where it was evident that the ERS inhibition by 4PBA leads to significant apoptosis induction. Programmed cell death is an important mechanism that leads to collateral destruction of cancer cells with occasional killing of normal cells and without an inflammatory response, which leads to the maintenance of proper homeostasis. Therefore, an apoptosis-inducing compound is beneficial as a useful chemotherapeutic agent against carcinogenic effects [35]. The ERS inhibition (4PBA alone) exhibited early apoptosis, which was evident by immune fluorescence and also the flow cytometry where the cell population was shifted from viable to apoptotic in the 4PBA alone treated group. This is comparable to a previous study by Kwan et al. on MCF-7 breast cancer cells [36].

**Figure 10:**
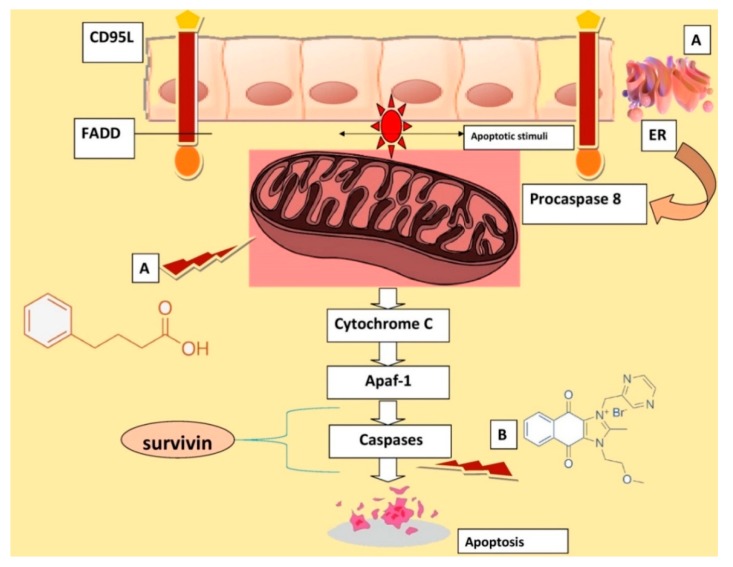
Mechanism and role of 4PBA and YM155. (**A**) 4PBA. (**B**) YM155.ERS is a distress signal triggered due to accumulation of misfolded proteins in the ER. 4PBA (**A**), which is an ERS inhibitor in our study, played a role in inhibiting the *UPR* genes by acting as an agent and by reducing the protein misfolding in the ER and also aiding in apoptosis via cytochrome-c release. The post-apoptotic signaling, Survivin, is released from the mitochondria block cytochrome-c and inhibits the Caspases, which inhibits programmed cell death. (**B**) YM155, which is a Survivin inhibitor, was found to escalate apoptosis via the caspase3/7 pathway and aiding the free flow release of Caspases and facilitating cell death.

Although Caspase 9 is a trigger of the intrinsic death pathway, Caspases 3 and 7 are regarded as the major effectors for apoptosis execution [36]. There are numerous roles played by Caspase 3 including apoptosis-associated chromatin margination and DNA fragmentation [37]. Our results showed that the concentration-dependent 4PBA increasedcaspase-3/7 activity in LS174T cells, which suggests that ERS inhibition by 4PBA-induced apoptosis is a Caspase-dependent mechanism (Figure 10), as induced in a previous study where the breast cancer cells were treated by plant extracts [38].

PARP activation and subsequent cleavage play a major role in programmed cell death [39]. 4PBA via ERS inhibition in our study could induce the cleavage of PARP, which is regarded as the hallmark of apoptosis [40]. Previous similar studies have reported a similar condition of calcium-dependent protease and calpain being activated in various models of apoptotic cell death, which results in atypical PARP cleavage [41]. Cancer cells standout from normal cells in aspects such as being proliferative, being anaerobic, and creating a microenvironment that is deprived of nutrients, which makes them resistant to cancer therapy [42]. This microenvironment also triggers ERS leading to apoptosis, but cancer cells have adapted themselves against the UPR alterations to avoid cell death. In our study, we have unraveled the connection between the changes in UPR and proliferation. The ERS induced by TUN escalated the proliferation rate in our study similar to a previous study where bleomycin activated ER stress-associated proteins including *Grp78*, *CHOP*, and ATF-4 both in vitro and in vivo, which increased the lung fibroblast proliferation via upstream activation of ERS by PI3K/AKT [43]. 4PBA could markedly reduce the proliferation rate (4PBA alone and 4PBA+TUN). The surprising decrease of proliferation by ERS inhibition paves the way for the therapeutic potential. UPR activation can enable cancer cells to survive and adapt to harsh environments, which indicates the example of ER stress being a key secondary event in melanoma development and which contributes to the resistance of apoptosis through the persistent expression of pro-survival instead of pro-apoptotic proteins [44]. It was no surprise that the sixth hour time point had the highest proliferation recorded where the rate of relative expression of *Grp78* and Survivin was the highest. The levels of Mucin2 (*Muc2*) is highly controversial in cancers especially colon and colorectal cancers. Patients with advanced mucinous colorectal carcinomas had a strong expression of *Muc2* with increased metastasis [45] and it has been correlated with another proliferative protein known as Galectin-3, which is highly involved in migration and survival. *Muc2* levels in the TUN-treated group skyrocketed when compared to the treated groups (4PBA and 4PBA+TUN). These treated groups were similarly shown in a study by Lakshmanan et al. where mucins were associated with poor prognosis in terms of lung cancer [46] and *Muc2* over-expression was found to be an independent prognostic factor for ovarian cancer patients [47]. ERS reduction significantly curtailed the *Muc2* expression, which indicated a clear anti-proliferative potential observed in our study.

YM155 was proved to act as metastasis antagonism in a mouse model of triple negative breast cancer [48]. YM155 was also utilized in a single-center study alongside docetaxel in patients with advanced hormone refractory prostate cancer and other tumors showed a partial positive response. A similar response was observed in patients with melanoma [49]. We initially assayed the effects of YM155 on LS174T cells treated with TUN and YM155 alone and noticed that YM155 could reduce the gene expression and protein expression of UPR genes in parallel to reducing Survivin expression along with the reduction in pro-inflammatory markers such asIL8 and IL4. Wagner et al. have revealed that YM155 played a pivotal role in reducing STAT3 signaling and UPR and was also used in the clinical evaluation of YM155 in patients with multiple myeloma [50]. There is a major regulation of apoptotic cell death by Caspases especially downstream Caspases such as 3 and 7. IAP proteins such as Survivin apart from controlling the Caspase activation platform also aid in cell survival via NF-κB signaling [51]. In line with previous findings by Wagner et al. where they have shown the pro-apoptotic potential of YM155 on myeloma cells [50] and head neck squamous cell carcinoma tissues [52], we found that YM155 could elevate the levels of Caspase 3/7 in the LS174T cell line in both the TUN and YM155 alone groups (Figure 10). With our findings, we can attest 4PBA and YM155 as therapeutic agents in colorectal cancer via ERS mediated effects. It would be appropriate to evaluate the above effects in a mouse model of colorectal cancer and observe the downstream effects.

## 5. Conclusions

In conclusions, this study, for the first time, unfolds the interconnection between ERS and Survivin and proves that they are directly related and reciprocate effects like apoptosis and inflammation. The study has also exposed that ERS induction has a negative effect including worsening of carcinogenicity and proliferation while inhibition has a profound effect on apoptosis enhancement and proliferation reduction in LS174T cells by 4PBA and YM155, which clearly depicts a therapeutic scope. We have also revealed that Survivin is a key molecule that links ERS and apoptosis.

## Supplementary Materials

The following are available online at http://www.mdpi.com/2073-4409/7/10/171/s1, Figure S1: mRNA expression of IRE1, FigureS2: Pathway representing IRE1 orchestrating XBP1.

## References

[1] Xu C., Bailly-Maitre B., Reed J.C. (2009). Endoplasmic reticulum stress: Cell life and death decisions. J. Clin. Investig..

[2] Wang W.A., Groenendyk J., Michalak M. (2014). Endoplasmic reticulum stress associated responses in cancer. Biochim. Biophys. Acta.

[3] Mc Guckin M.A., Eri R.D., Das I., Lourie R., Florin T.H. (2010). ER stress and the unfolded protein response in intestinal inflammation. Am. J. Physiol. Gastrointest. Liver Physiol..

[4] Ma Y., Hendershot L.M. (2004). ER chaperone functions during normal and stress conditions. J. Chem. Neuroanat..

[5] Giampietri C., Petrungaro S., Conti S., Facchiano A., Filippini A., Ziparo E. (2015). Cancer microenvironment and endoplasmic reticulum stress response. Mediators Inflamm..

[6] Silke J., Meier P. (2013). Inhibitor of Apoptosis (IAP) Proteins–Modulators of Cell Death and Inflammation. Cold Spring Harb. Perspect. Biol..

[7] Altznauer F., Martinelli S., Yousefi S., Thürig C., Schmid I., Conway E.M., Schöni M.H., Vogt P., Mueller C., Fey M.F. (2004). Inflammation-associated cell cycle-independent block of apoptosis by survivin in terminally differentiated neutrophils. J. Exp. Med..

[8] Martini E., Wittkopf N., Günther C., Leppkes M., Okada H., Watson A.J., Podstawa E., Backert I., Amann K., Neurath M.F. (2016). Loss of Survivin in intestinal epithelial progenitor cells leads to mitotic catastrophe and breakdown of gut immune homeostasis. Cell. Rep..

[9] Qi G., Kudo Y., Tang B., Liu T., Jin S., Liu J., Zuo X., Mi S., Shao W., Ma X. (2016). PARP6 acts as a tumor suppressor via downregulating Survivin expression in colorectal cancer. Oncotarget.

[10] Cubillos-Ruiz J.R., Bettigole S.E., Glimcher L.H. (2017). Tumorigenic and immunosuppressive effects of endoplasmic reticulum stress in cancer. Cell.

[11] Farooqi A.A., Li K.T., Fayyaz S., Chang Y.T., Ismail M., Liaw C.C., Yuan S.S., Tang J.Y., Chang H.W. (2015). Anticancer drugs for the modulation of endoplasmic reticulum stress and oxidative stress. Tumour Biol..

[12] Mahadevan N.R., Rodvold J., Sepulveda H., Rossi S., Drew A.F., Zanetti M. (2011). Transmission of endoplasmic reticulum stress and pro-inflammation from tumor cells to myeloid cells. Proc. Natl. Acad. Sci. USA.

[13] Heazlewood C.K., Cook M.C., Eri R., Price G.R., Tauro S.B., Taupin D., Thornton D.J., Png C.W., Crockford T.L., Cornall R.J. (2008). Aberrant mucin assembly in mice causes endoplasmic reticulum stress and spontaneous inflammation resembling ulcerative colitis. PLoS. Med..

[14] Tawiah A., Cornick S., Moreau F. (2018). High *Muc2* mucin expression and misfolding induce cellular stress, reactive oxygen production, and apoptosis in goblet cells. Am. J. Pathol..

[15] Jang T.J., Cho M.Y. (2005). Cyclooxygenase-2 expression and cell proliferation are increased in *Muc2*-positive area of columnar-lined esophagus. Pathol. Int..

[16] Hernandez J.M., Farma J.M., Coppola D., Hakam A., Fulp W.J., Chen D.T., Siegel E.M., Yeatman T.J., Shibata D. (2011). Expression of the antiapoptotic protein survivin in colon cancer. Clin. Colorectal. Cancer.

[17] Mita A.C., Mita M.M., Nawrocki S.T., Giles F.J. (2008). Survivin: Key regulator of mitosis and apoptosis and novel target for cancer therapeutics. Clin. Cancer Res..

[18] Sano R., Reed J.C. (2013). ER stress-induced cell death mechanisms. Biochim. Biophys. Acta.

[19] Chen W.C., Liu Q., Fu J.X., Kang S.Y. (2004). Expression of survivin and its significance in colorectal cancer. World J. Gastroenterol..

[20] Jayakumar J., Anishetty S. (2014). Molecular dynamics simulations of inhibitor of apoptosis proteins and identification of potential small molecule inhibitors. Bioorg. Med. Chem. Lett..

[21] Yadav R.K., Chae S.W., Kim H.R., Chae H.J. (2014). Endoplasmic reticulum stress and cancer. J. Cancer Prev..

[22] Kim H.J., Jeon J.S., Kim H.R., Park S.Y., Chae H.J., Lee Y.C. (2013). Inhibition of endoplasmic reticulum stress alleviates lipopolysaccharide-induced lung inflammation through modulation of NF-κB/HIF-1α signaling pathway. Sci. Rep..

[23] Waugh D.J., Wilson C. (2008). The interleukin-8 pathway in cancer. Clin. Cancer Res..

[24] Chen L., Fan J., Chen H., Meng Z., Chen Z., Wang P., Liu L. (2014). The IL8/CXCR1 axis is associated with cancer stem cell-like properties and correlates with clinical prognosis in human pancreatic cancer cases. Sci. Rep..

[25] Koller F.L., Hwang D.G., Dozier E.A., Fingleton B. (2010). Epithelial interleukin-4 receptor expression promotes colon tumor growth. Carcinogenesis.

[26] Todaro M., Perez Alea M., Scopelliti A., Medema J.P., Stassi G. (2008). IL4-mediated drug resistance in colon cancer stem cells. Cell. Cycle.

[27] Francipane M.G., Alea M.P., Lombardo Y., Todaro M., Medema J.P., Stassi G. (2008). Crucial role of interleukin-4 in the survival of colon cancer stem cells. Cancer Res..

[28] Di Stefano A.B., Iovino F., Lombardo Y., Eterno V., Höger T., Dieli F., Stassi G., Todaro M. (2010). Survivin is regulated by interleukin-4 in colon cancer stem cells. J. Cell. Physiol..

[29] Oft M. (2014). IL10: Master switch from tumor-promoting inflammation to antitumor immunity. Cancer Immunol. Res..

[30] Purushottam L., Lavakumar K., Barath S., Keith K. (2015). PD-1 and IL-10: Partners in crime against anti-tumor immunity in ovarian cancer. J. Immunol..

[31] Waldner M.J., Foersch S., Neurath M.F. (2012). Interleukin-6—A key regulator of colorectal cancer development. Int. J. Biol. Sci..

[32] Nagasaki T., Hara M., Nakanishi H., Takahashi H., Sato M., Takeyama H. (2014). Interleukin-6 released by colon cancer-associated fibroblasts is critical for tumour angiogenesis: Anti-interleukin-6 receptor antibody suppressed angiogenesis and inhibited tumour-stroma interaction. Br. J. Cancer.

[33] Gerl R., Vaux D.L. (2005). Apoptosis in the development and treatment of cancer. Carcinogenesis.

[34] Virrey J.J., Dong D., Stiles C., Patterson J.B., Pen L., Ni M., Schontal A.H., Chen T.C., Hofman F.M., Lee A.S. (2008). Stress chaperone GRP78/BiP confers chemoresistance to tumor-associated endothelial cells. Mol. Cancer Res..

[35] Badmus J.A., Ekpo O.E., Hussein A.A., Meyer M., Hiss D.C. (2015). Antiproliferative and apoptosis induction potential of the methanolic leaf extract of *Holarrhena floribunda* (G. Don). Evid. Based Complement. Altern. Med..

[36] Kwan Y.P., Saito T., Ibrahim D., Al-Hassan F.M., Ein Oon C., Chen J., Jothy S.L., Kanwar J.L., Sasidharan S. (2016). Evaluation of the cytotoxicity, cell-cycle arrest, and apoptotic induction by Euphorbia hirta in MCF-7 breast cancer cells. Pharm. Biol..

[37] Slee E.A., Adrain C., Martin S.J. (2001). Executioner caspase-3, -6, and -7 perform distinct, non-redundant roles during the demolition phase of apoptosis. J. Biol. Chem..

[38] Looi C.Y., Arya A., Cheah F.K., Muharram B., Leong K.H., Mohamad K., Wong W.F., Rai N., Mustafa M.R. (2013). Induction of apoptosis in human breast cancer cells via caspase pathway by vernodalin isolated from *Centratherumanthelminticum* (L.) seeds. PLoS ONE.

[39] Boulares A.H., Yakovlev A.G., Ivanova V., Stoica B.A., Wang G., Iyer S., Smulson M. (1999). Role of poly(ADP-ribose) polymerase (PARP) cleavage in apoptosis. Caspase 3-resistant PARP mutant increases rates of apoptosis in transfected cells. J. Biol. Chem..

[40] Brauns S.C., Dealtry G., Milne P., Naudé R., Van de Venter M. (2005). Caspase-3 activation and induction of PARP cleavage by cyclic dipeptide cyclo(Phe-Pro) in HT-29 cells. Anticancer Res..

[41] Pink J.J., Wuerzberger-Davies S., Tagliarino C., Planchon S.M., Yang X., Froelich C.J., Boothman D.A. (2000). Activation of a cysteine protease in MCF-7 and T47D breast cancer cells during ‚-lapachone-mediated apoptosis. Exp. Cell. Res..

[42] Holst S., Belo A.L., Giovannetti E., van Die I., Wuhrer M. (2017). Profiling of different pancreatic cancer cells used as models for metastatic behaviour shows large variation in their N-glycosylation. Sci. Rep..

[43] Hsu H.S., Liu C.C., Lin J.H., Hsu T.W., Hsu J.W., Su K., Hung S.C. (2017). Involvement of ER stress, *PI3K*/*AKT* activation, and lung fibroblast proliferation in bleomycin-induced pulmonary fibrosis. Sci. Rep..

[44] Corazzari M., Gagliardi M., Fimia G.M., Piacentini M. (2017). Endoplasmic reticulum stress, unfolded protein response, and cancer cell fate. Front. Oncol..

[45] Song S., Byrd J.C., Mazurek N., Liu K., Koo J.S., Bresalier R.S. (2005). Galectin-3 modulates *Muc2* mucin expression in human colon cancer cells at the level of transcription via AP-1 activation. Gastroenterology.

[46] Lakshmanan I., Ponnusamy M.P., Macha M.A., Haridas D., Majhi P.D., Kaur S., Jain M., Batra S.K., Ganti A.K. (2015). Mucins in lung cancer: Diagnostic, prognostic, and therapeutic implications. J. Thorac. Oncol..

[47] He Y.F., Zhang M.Y., Wu X., Sun X.J., Xu T., He Q.Z., Di W. (2013). High *Muc2* expression in ovarian cancer is inversely associated with the M1/M2 ratio of tumor-associated macrophages and patient survival time. PLoS ONE.

[48] Yamanaka K., Nakata M., Kaneko N., Fushiki H., Kita A., Nakahara T., Koutoku H., Sasamata M. (2011). YM155, a selective survivin suppressant, inhibits tumor spread and prolongs survival in a spontaneous metastatic model of human triple negative breast cancer. Int. J. Oncol..

[49] Lewis K.D., Samlowski W., Ward J., Catlett J., Cranmer L., Kirkwood J., Lawson D., Whitman E., Gonzalez R. (2011). A multi-center phase II evaluation of the small molecule Survivin suppressor YM155 in patients with unresectable stage III or IV melanoma. Investig. New Drugs.

[50] Wagner V., Hose D., Seckinger A., Weiz L., Meiβner T., Rème T., Breitkreutz I., Podar K., Ho A.D., Goldschmidt H. (2014). Preclinical efficacy of sepantronium bromide (YM155) in multiple myeloma is conferred by down regulation of Mcl-1. Oncotarget.

[51] Oberoi-khanuja T.K., Murali A., Rajalingam K. (2013). IAPs on the move: Role of inhibitors of apoptosis proteins in cell migration. Cell Death Dis..

[52] Zhang L., Zhang W., Wang Y.F., Liu B., Zhang W.F., Zhao Y.F., Kulkarni A.B., Sun Z.J. (2016). Dual induction of apoptotic and autophagic cell death by targeting survivin in head neck squamous cell carcinoma. Cell Death Dis..

