# Supplementary material for: Interplay between Endoplasmic Reticular Stress and Survivin in Colonic Epithelial Cells
Source: Cells. 2018 Oct 15;7(10):171. doi: 10.3390/cells7100171 (PMC6210275; doi:10.3390/cells7100171)
Supplement: Supplementary file 1 [file cells-07-00171-s001.pdf]

Interplay Between Endoplasmic Reticular Stress and Survivin in Colonic Epithelial Cells

Rohit Gundamaraju *, Ravichandra Vemuri, Wai Chin Chong, Stephen Myers, Shaghayegh Norouzi, Madhur D. Shastri and Rajaraman Eri *

School of Health Sciences, University of Tasmania, Launceston, Tasmania, Australia 7248; ravichandra.vemuri@utas.edu.au (R.V.); chongwc1993@gmail.com (W.C.C.); Stephen.myers@utas.edu.au (S.M.); shaghayeg.norouzi@utas.edu.au (S.N.); madhur.shastri@utas.edu.au (M.D.S.)

* Correspondence: rohit.gundamaraju@utas.edu.au (R.G.); rderi@utas.edu.au (R.E.);

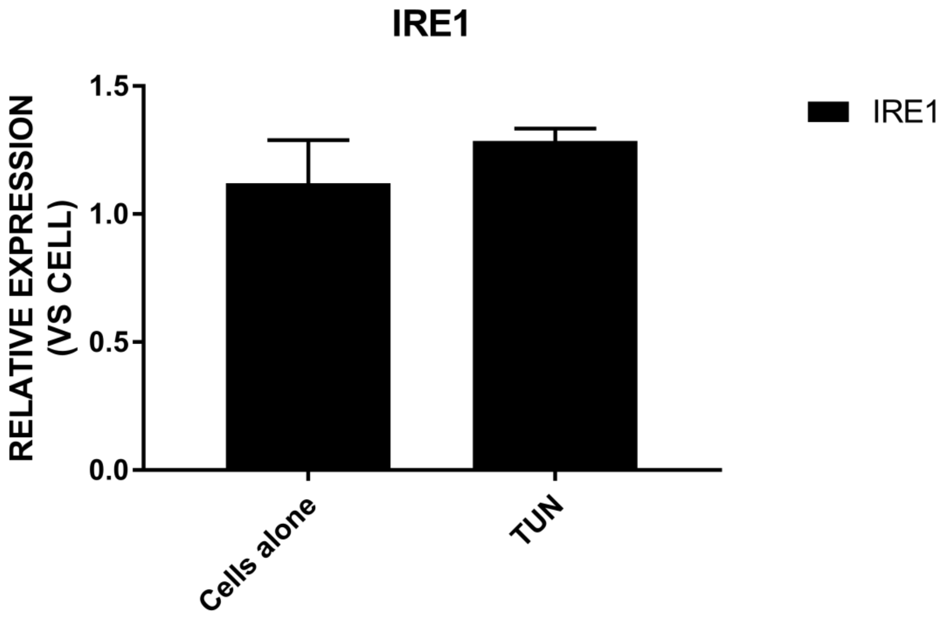

Supplementary figure1: IRE1 mRNA expression upon TUN induction

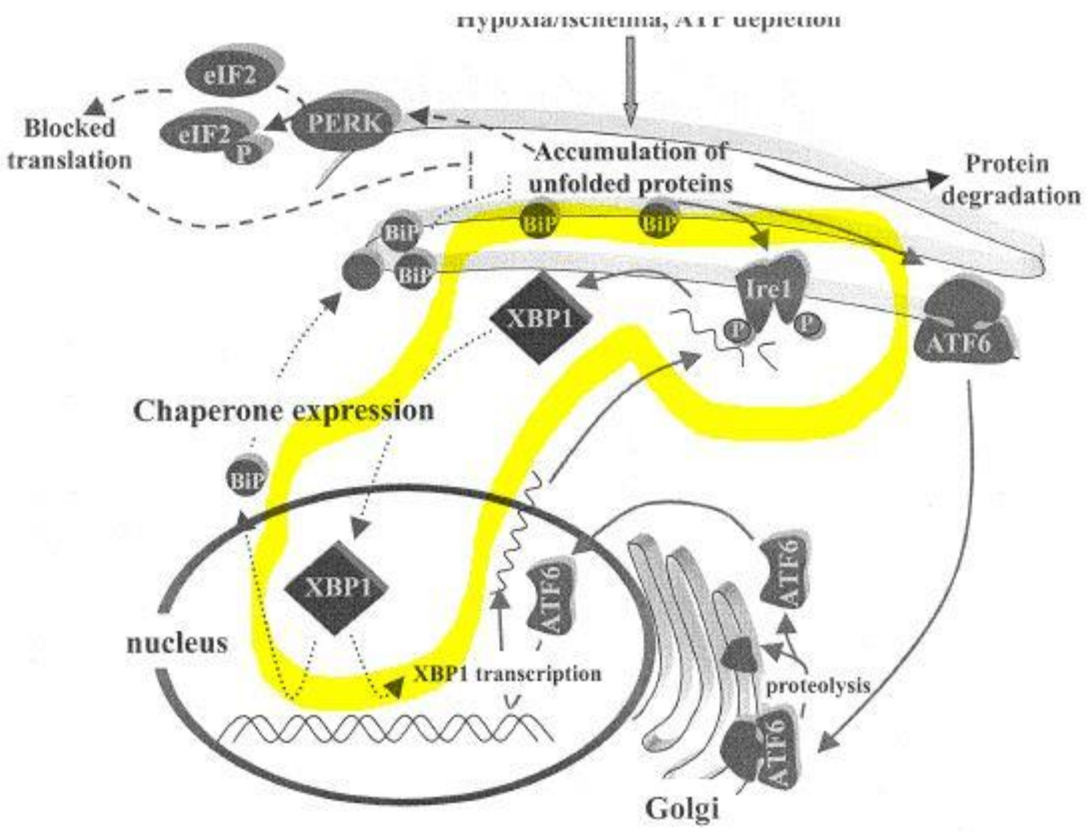

Supplementary figure2: Pathway of IRE1 to XBP1 under stress
